# Rugged Large Volume Injection for Sensitive Capillary LC-MS Environmental Monitoring

**DOI:** 10.3389/fchem.2017.00062

**Published:** 2017-08-28

**Authors:** Hanne Roberg-Larsen, Silvija Abele, Deniz Demir, Diana Dzabijeva, Sunniva F. Amundsen, Steven R. Wilson, Vadims Bartkevics, Elsa Lundanes

**Affiliations:** ^1^Department of Chemistry, University of Oslo Oslo, Norway; ^2^Faculty of Chemistry, University of Latvia Riga, Latvia

**Keywords:** capillary LC, column switching, large volume injection, on-line solid phase extraction, pharmaceutical products

## Abstract

A rugged and high throughput capillary column (cLC) LC-MS switching platform using large volume injection and on-line automatic filtration and filter back-flush (AFFL) solid phase extraction (SPE) for analysis of environmental water samples with minimal sample preparation is presented. Although narrow columns and on-line sample preparation are used in the platform, high ruggedness is achieved e.g., injection of 100 non-filtrated water samples did not result in a pressure rise/clogging of the SPE/capillary columns (inner diameter 300 μm). In addition, satisfactory retention time stability and chromatographic resolution were also features of the system. The potential of the platform for environmental water samples was demonstrated with various pharmaceutical products, which had detection limits (LOD) in the 0.05–12.5 ng/L range. Between-day and within-day repeatability of selected analytes were <20% RSD.

## Introduction

An increasing consumption of pharmaceutical products (PPs) raises significant concerns, as their growing presence in the environment can represent a serious threat to our health and the environment (Khetan and Collins, 2007; Pailler et al., 2009; Kumar et al., 2010; Fatta-Kassinos et al., 2011; Hoff et al., 2015; Richardson and Kimura, 2016). For example, the continuous presence of pharmaceuticals and their metabolites in water bodies (Khetan and Collins, 2007), even at very low concentrations (low ng/L, Fatta-Kassinos et al., 2011; Houtman et al., 2014; Richardson and Kimura, 2016), can possibly lead to unwanted biological effects on aquatic species (Kumar et al., 2010; Fatta-Kassinos et al., 2011; Houtman et al., 2014; Richardson and Kimura, 2016), such as antibiotic resistance (Garcia-Ac et al., 2009; Kumar et al., 2010; Houtman et al., 2014; Richardson and Kimura, 2016; Tetzner et al., 2016) and other ecotoxicological effects (Garcia-Ac et al., 2009; Fatta-Kassinos et al., 2011; Richardson and Kimura, 2016). Thus, ultra-trace PP monitoring is an important task (Houtman et al., 2014), but has considerable analytical challenges (Kumar et al., 2010; Fatta-Kassinos et al., 2011), even for today's state-of-the-art liquid chromatography-mass spectrometry (LC-MS) systems. Hence, substantial sample preparation approaches, often requiring manual handling, are used for analyte enrichment (Garcia-Ac et al., 2009; Richardson and Kimura, 2016). One can however modify LC-MS systems to increase sensitivity, e.g., by using very narrow LC columns (inner diameter <0.5 mm). Columns with reduced inner diameter have less chromatographic dilution (radial) (Chervet et al., 1996; Vissers et al., 1997), allowing increased sensitivity when using concentration sensitive detectors such as electrospray ionization (ESI) MS (Chervet et al., 1996; Dear et al., 1999; Vissers, 1999; Shen et al., 2002; Wilson et al., 2015). Detection limits can be further improved by injecting large volumes using column switching systems (Vissers et al., 1996; Wilson et al., 2007; Rogeberg et al., 2014). Narrow capillary LC (cLC) columns and switching systems are arguably mostly associated with proteomics, although the approach has been used in e.g., pharmaceutical analysis as well (Ayrton et al., 1999; Dear et al., 1999; Granger et al., 2005). However, the large volume injection/column switching/cLC approach has also potential in environmental analysis, especially when target analytes are present at very low concentrations. The cLC approach allows for less sample to be collected/handled, and this has benefits regarding time spent on sample preparation (Garcia-Ac et al., 2009), and simpler sample transport/storage (mL vs. L, Stravs et al., 2016). When sample preparation is automated, repeatability can be improved and sources of error are reduced (Garcia-Ac et al., 2009; Fatta-Kassinos et al., 2011; Tetzner et al., 2016). Although LC-MS with column switching (Stoob et al., 2005; Pozo et al., 2006; Garcia-Ac et al., 2009; Singer et al., 2010; Stravs et al., 2016; Tetzner et al., 2016) has have been used in environmental monitoring, the large volume injection/column switching/cLC approach has not been widely embraced (Stravs et al., 2016). A core concern regarding column switching and cLC is reduced ruggedness, much due to clogging of columns and connections. However, we have developed a plumbing scheme that allows for greatly improved column switching/cLC ruggedness, called automatic filtration and filter back-flush (AFFL) (Svendsen et al., 2011; Roberg-Larsen et al., 2014; Johnsen et al., 2015). AFFL features a simple self-cleaning filter system, which has been used for a variety of bioanalytical applications (Røen et al., 2014; Johnsen et al., 2015; Brandtzaeg et al., 2016; Roberg-Larsen et al., 2017), but not yet for environmental applications. We here demonstrate the potential of the 10-port switching valve AFFL-SPE-cLC system for environmental analytical chemistry with creek water as matrix.

## Materials and methods

### Chemicals and reagents

All chemicals and reagents were of analytical grade or higher. All analytes and isotope-labeled internal standards (IS) (See Table S1) were from Sigma Aldrich (St. Louis, MO, USA). Mobile phase A consisted of 0.1% formic acid (100%, Sigma Aldrich) in type 1 water (Milli-Q water purification system, Millipore, Bedford, USA) while mobile phase B was 0.1% formic acid in methanol (MeOH, VWR, Radnor, PA, USA).

### Standards solutions and samples

All equipment used (e.g., balance and pipettes) in preparation of stock solutions, working solutions and standard solutions were newly calibrated. Stock solutions were made by dissolving analytes and sulfamethoxazole-(phenyl^13^C_6_) internal standard in 0.1% formic acid in MeOH to 1 mg/mL. Atenolol-d7 internal standard was dissolved in acetonitrile + MeOH (8+2) in concentration 0.4 mg/mL. These stock solutions were diluted with appropriate volumes of 0.1% formic acid in type 1 water to give 100 ng/L working solutions.

Standard solutions were made by spiking either type 1 water or non-filtrated creek water samples from Alnaelva and Blindernbekken (both Oslo) with appropriate volumes to give concentration in the range of 0.05–50 ng/L. All standard solutions contained 10 ng/L internal standard. Limit of detection (LOD) was defined as the concentration giving a signal to noise ratio > 3 (*n* = 3). LOQ was here defined as the lowest concentration that gives a repeatable peak area (RSD < 20%, *n* = 3). The compounds investigated (see Table S1) are fairly water-soluble. If more hydrophobic compounds were to be determined, additional extraction steps may be necessary (e.g., extraction from particular matter present in the water matrix).

Repeatability and recovery (for selected analytes; trimethoprim, sulfapyridine, sulfamethoxazole, and atenolol) were examined by spiking standard solutions into non-filtrated creek water samples from Sognsvannsbekken (Oslo) in concentrations 10 ng/L (low), 50 ng/L (medium), and 100 ng/L (high). Atenolol-d_7_ was used as an internal standard for atenolol, while sulfamethoxazole-(phenyl-^13^C_6_) was used as an internal standard for the other analytes. Within-day (*n* = 6) and between-day (*n* = 3) repeatabilities were examined using analysis of variance (ANOVA) in Excel. Apparent recovery was calculated by comparing the signal/ concentration slope of spiked creek water with that of spiked type 1 water.

$$\begin{array}{l} Apparant\;recovery\;\left(\%\right)=\frac{slope\;spiked\;creek\;water}{slope\;spiked\;type\;1\;water}x100\;\% \end{array}$$

### Instrumental setup

**Figure 1:**
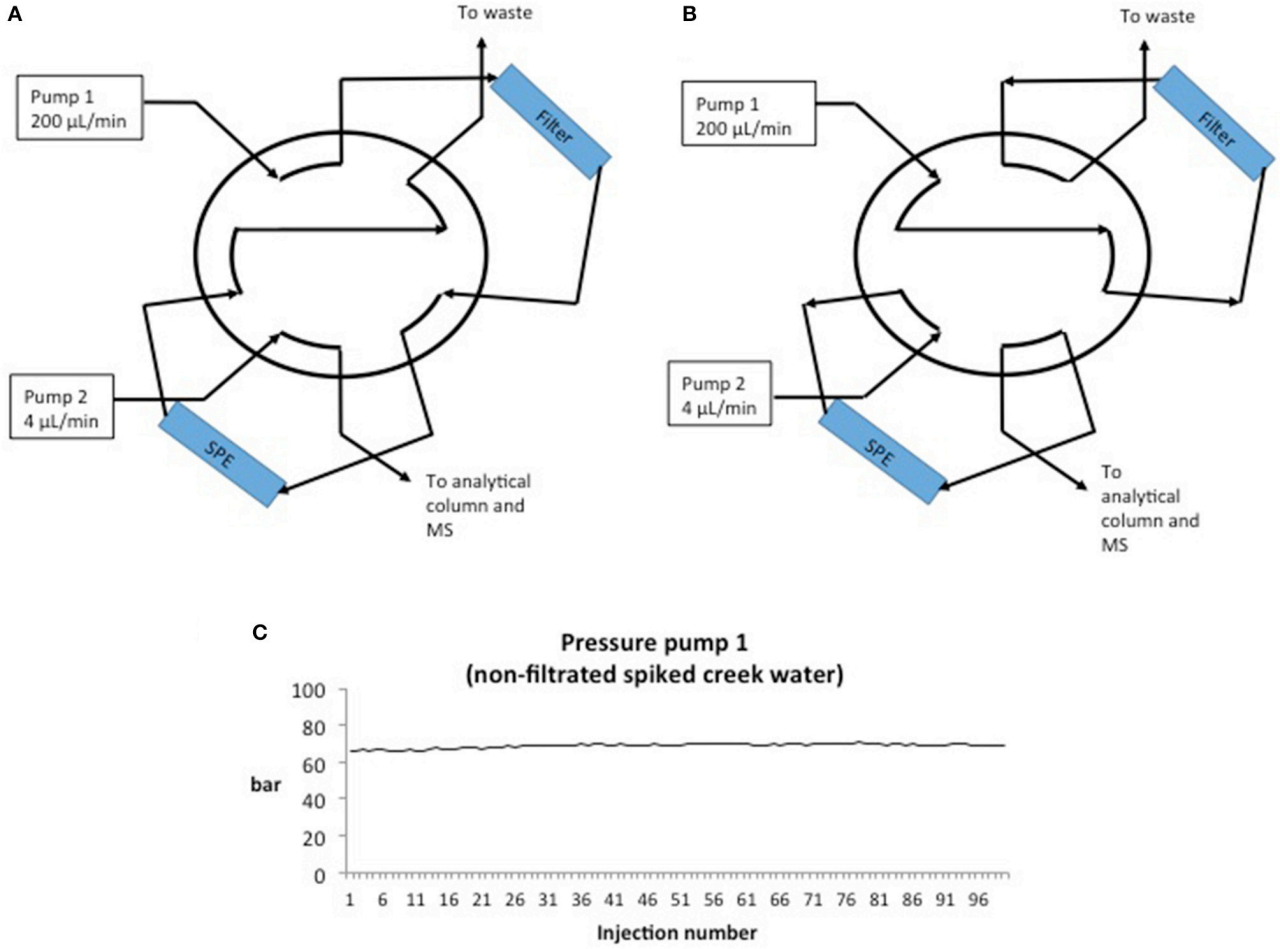
**(A)** AFFL-SPE in load position. Solutions (100 μL) are injected by an autosampler and transferred through the filter and on to the SPE column by Pump 1 (200 μL/min). Particles are stopped by the filter, while analytes are trapped on the SPE column. **(B)** AFFL-SPE in inject position. Pump 2 (4 μL/min) elutes analytes from SPE and on to the analytical column for separation and MS for detection. Simultaneously, pump 1 back-flushes the filter, washing particles out to waste. **(C)** Pressure on the SPE column/pump 1 in inject position while injecting 100 non-filtrated spiked creek water samples.

A schematic view of the AFFL-SPE-cLC-MS system is shown in Figure 1. Standard solutions and spiked creek water samples were injected (100 μL) by an Agilent G1313A ALS autosampler on to the 10 port, two position switching valve (1/16″, 0.25 mm bore, Valco, Houston, TX, USA) using mobile phase A (150 μL/min) delivered by an Agilent G1310A IsoPump pump (Figure 1A). The samples and standard solutions were filtrated on-line through a 1 μm stainless steel filter/screen (Valco). Analytes were trapped on-line on a HotSep C_18_ SPE column (3 μm particles, 1 mm ID × 50 mm) from G&T Septech (Ski, Norway). The 10-port switching valve, autosampler and pumps were controlled by Chemstation version B.04.03, and after 1 min the valve was switched to position 2 (Figure 1B). Simultaneously, pump 2 (Agilent G1376A CapPump) eluted the analytes off the SPE column, while pump 1 back-flushed the filter. The analytes were separated on a HotSep C_18_ column, (G&T Septech, 2 μm particles, 0.3 mm ID × 150 mm) or an ACE 3 C18 column (Advanced Chromatography Technologies, 3 μm particles, 0.3 mm ID × 150 mm, for repeatability and recovery experiments) using a 7 min solvent gradient from 35 to 95% B (7 min duration). The mobile phase was then held at 95% B for 7 min to wash out more hydrophobic compounds originating from the water samples before lowering the % B to starting conditions in 1 min. The LC flow rate was 4 μL/min (as recommended by the manufacturer of the column), but could likely be increased if a UHPLC pump were to be used. Column reconditioning was performed while the next standard solution or sample was loaded on to the SPE column. The total analysis time, including sample loading and reconditioning, was 15 min.

The column outlet was coupled to a high-resolution mass spectrometer (QExactive Orbitrap, Thermo Scientific, Waltham, MA, USA) using a standard heated electrospray ionization source (H-ESI) operated in positive mode (+3,500 V). Sheath gas was set to 5, while auxiliary gas was 1. The source temperature was 50°C. The MS was controlled by Xcalibur software version 3.0 and was operated in target MS^2^ mode [equal to parallel reaction monitoring (PRM)]. Fragmentation energy and monitored ions are shown in Table S1 (Supplementary Material). Resolution was set to 35,000, with AGC target 2e5 and maximum injection time 100 ms. For recovery and repeatability studies, a TSQ Quantiva (Triple Q, Thermo Scientific) equipped with H-ESI source was employed, with settings as described above. The triple Q instrument was operated in SRM mode with fragmentation energy and monitored ions as shown in Table S1. CID gas was set to 0.5 mTorr and resolution was 0.2 and 04 Da for Q1 and Q3, respectively. Cycle time was set to 1.

## Results and discussion

### Ruggedness of pressure and retention time

**Figure 2:**
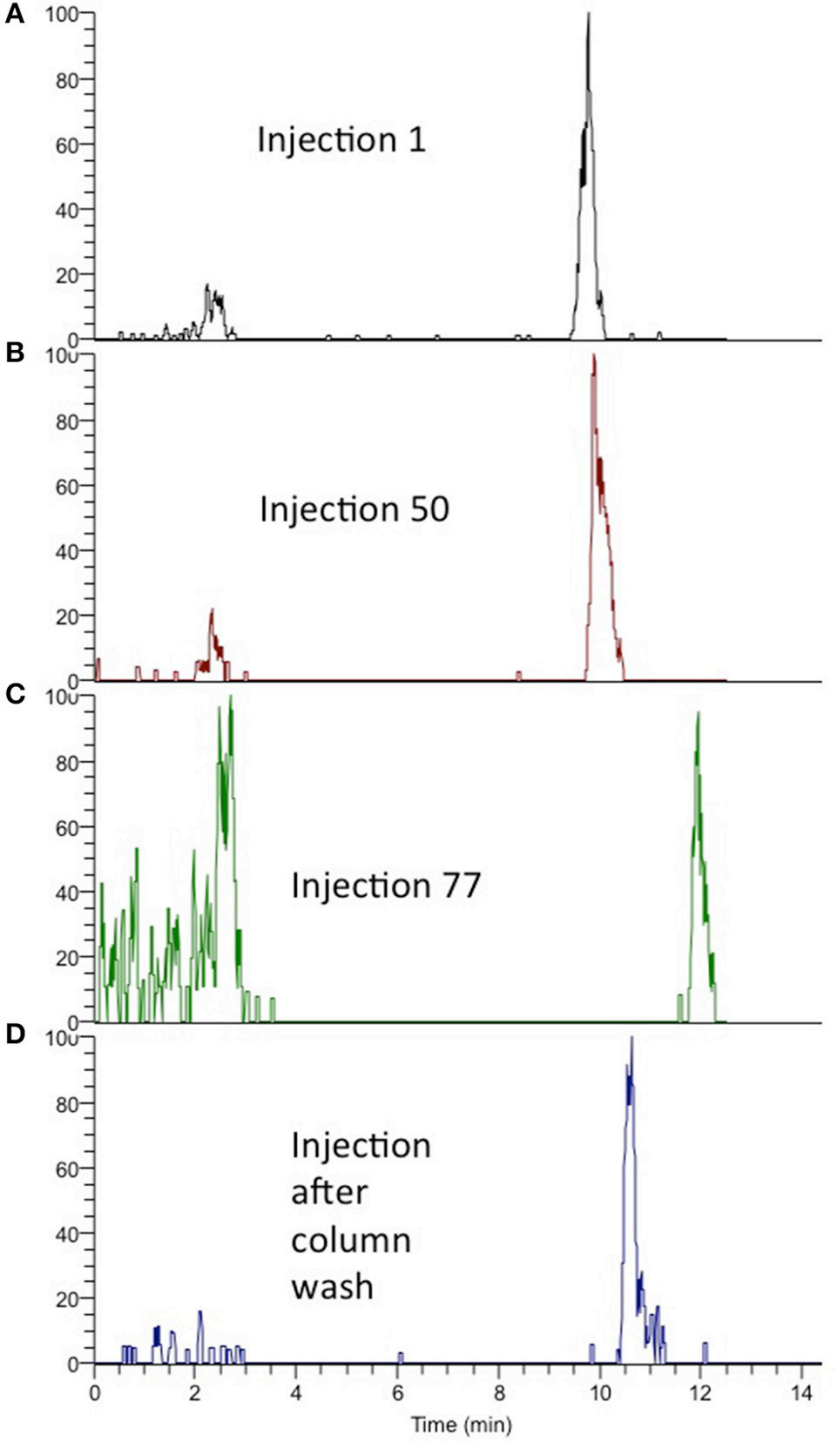
**(A)** Retention of sulfamethoxazole, injection no. 1. **(B)** Retention after 50 injections. **(C)** Retention after 77 injections. **(D)** Retention after washing procedure (95% MeOH).

A common concern with on-line SPE is that particles and precipitants from samples can easily clog the SPE columns, resulting in increased maintenance requirement and decreased ruggedness. Also, if the trapped analytes are back-flushed off the SPE column, these particles can be transferred to the analytical column, resulting in a clogged analytical column (as well). To avoid these problems, an off-line filtration (Stoob et al., 2005; Garcia-Ac et al., 2009; Singer et al., 2010; Tetzner et al., 2016) or SPE procedure is often included in the method, increasing manual steps and introduction of potential human errors (Majors, 1991; Chen et al., 2009; Stravs et al., 2016; Tetzner et al., 2016). Using the AFFL plumbing, these problems are avoided; particles are trapped by an on-line filter (stainless steel), while analytes are enriched on-line on an SPE column (Figure 1A) using a 10 port switching valve controlled by the LC pump, high flow loading pump (150 μL/min) and non-eluting conditions (0.1% formic acid in H_2_O). When analytes are eluted from the SPE column to the analytical column (Figure 1B), the filter is simultaneously back-flushed, making it clean and ready for the next sample. With this effort, ruggedness of the system is greatly improved and non-filtrated water samples can be injected without pressure rise/clogging of SPE or analytical column. A graph showing pressure over the SPE column during 100 injections (100 μL per injection) of non-filtrated, spiked creek water is presented in Figure 1C. The pressure on the SPE column (sample loading, pump 1) was constant during all injections (69 ±1 bar), confirming that no clogging of the SPE column had occurred. Analytes were back-flushed off the SPE column in elution mode (position 2, Figure 1). The back-pressure of the analytical column (pump 2) was also constant during the injections (data not shown), implying that no particles were transferred from the SPE column to the analytical column. The peak shape and retention of the analytes were similar for dozens of injections without column washing steps. Beyond about 60 injections, the retention times would increase gradually (as shown for injection 77 in Figure 2C), but a programmed column wash with 95% B restored retentions times again (Figure 2D). Thus, for the matrix tested (creek water), we recommend column washing after 50 injections (Figures 2A,B).

### Sensitivity and peak shape

**Figure 3:**
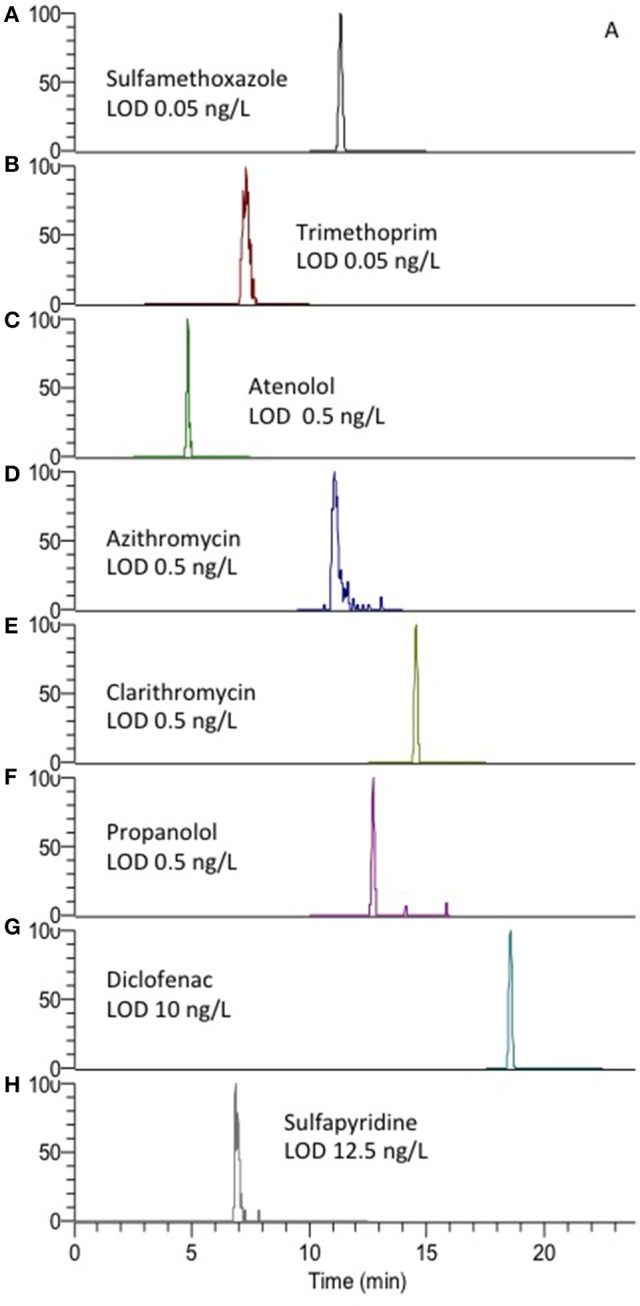
Extracted ion chromatograms (EICC) of **(A)** sulfamethoxazole, **(B)** trimethoprime, **(C)** atenolol, **(D)** azithromycin, **(E)** clarithromycin, **(F)** propanolol, **(G)** diclofenac, and **(H)** sulfapyridine using the described AFFL-SPE-LC-MS system. MS/MS transition details are presented in Table S1.

The rugged method with large volume injection in combination with narrow columns (cLC, ID 0.3 mm) and the concentration sensitive detector ESI-MS could be used to achieve detection limits in the low ng/L range as required. LODs were in the range 0.05–12.5 ng/L (Figure 3). LOQs were 1–12.5 ng/L for sulfamethoxazole and sulfapyridine (compounds of particular interest in our current research), respectively. Aliquots of 100 μL (maximum volume of the available autosampler) were needed to achieve limit of quantification (LOQ) in the low ng/L range. The LOQs are comparable to that of other methods where as much as 1 L water is subject to off-line SPE to enrich the sample for quantitative detection (Vanderford et al., 2003).

Chromatograms showing peaks at LOD for other possible PPs are also shown in Figure 3, which also shows that the peak shape could be satisfactory (asymmetry factors, A_s_, were 0.7–3.1, calculated at 10% peak height), implying that the system allows for low dead volumes/decent refocusing. Asymmetry (Table S2) of some compounds was however observed, especially for sulfapyridine (A_*s*_ 3.1), as also observed by others (Tetzner et al., 2016). Thus the high A_*s*_ may be considered to be compound dependent in light of the peak shapes of the other compounds.

**Figure 4:**
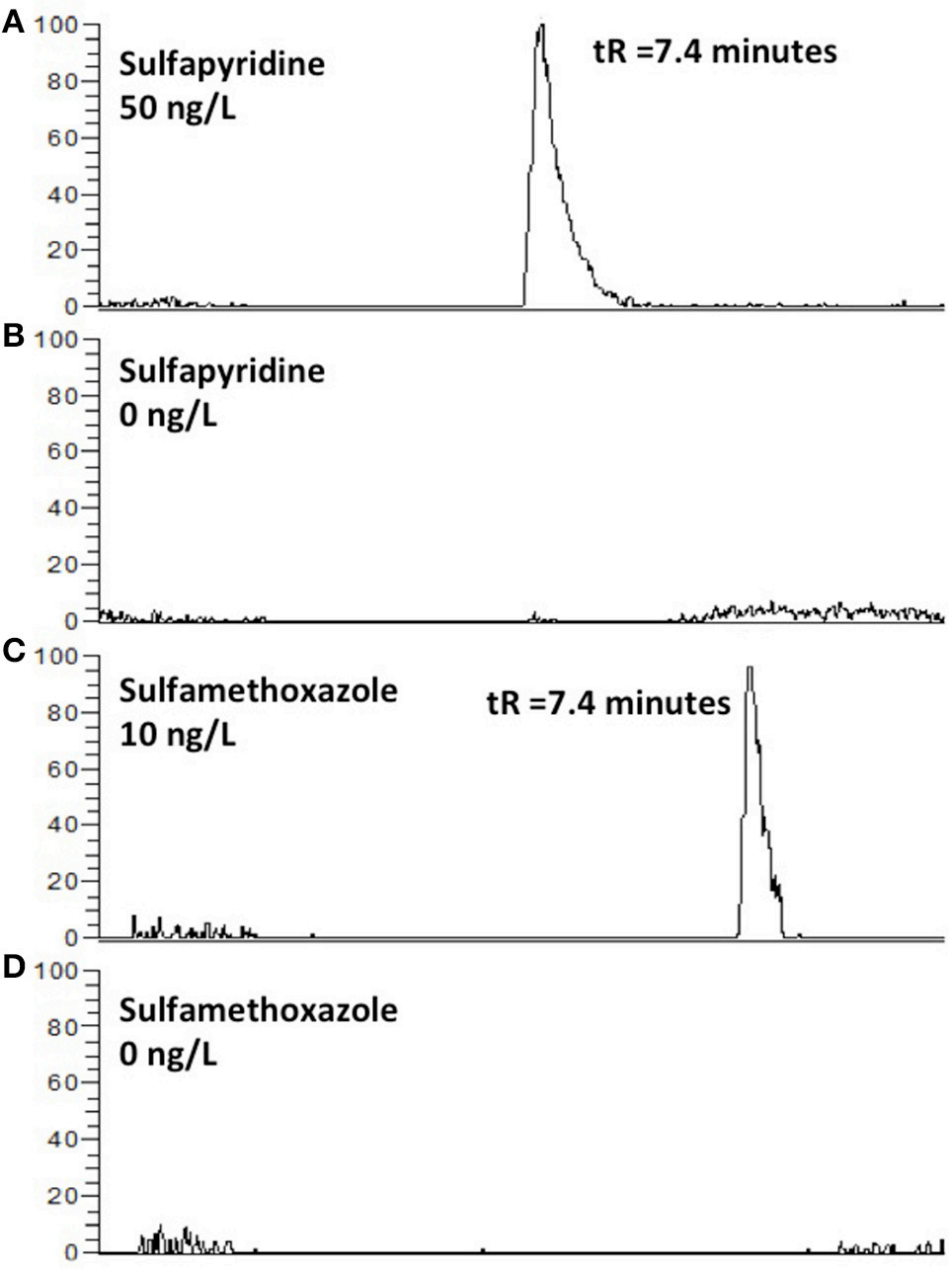
EICC of sulfapyridine **(A)**, sulfametoxazole **(C)** followed by an injection of type 1 water **(B,D)**. Carry-over was not observed.

Another concern with column switching systems is carry-over (Asakawa et al., 2007). This was however not an issue when applying the AFFL-SPE system for creek water; no carry-over (<<1%) was observed for e.g., sulfonamides (Figure 4). Hence, the equipment used in the set-up (described in detail in the Materials and Methods section) has few issues with adsorption, which is also highly advantageous when low, but reliable, detection limits are sought for (as those shown in Figure 3).

### Linearity, repeatability and apparent recovery

All selected analytes showed good linearity (*R*^2^ > 0.99) and good within day (RSD < 10%, *n* = 6) repeatability for all analytes spiked in both type 1 water and creek water, except sulfapyridine (spiked in creek water, RSD = 17%). All analytes showed good between day repeatability (RSD < 20%, *n* = 3), except for trimethoprim (RSD 46–125%), although the linearity of this compound each day was good (*R*^2^ = 0.99). These deviations can be explained by not available isotope-labeled standard for these two compounds at the time of study. Thus, although the cLC-based system provides excellent sensitivity and ruggedness, quantification issues can arise depending on the analyte (and interferences). Apparent recovery was between 81 and 151% for the analytes investigated (all data are shown in Table S3, Supplementary Material). These values imply varying degrees of ion suppression/enhancement, although miniaturized systems are associated with reduced matrix effects (Gosetti et al., 2010).

## Concluding remarks

A rugged and sensitive platform with minimal sample preparation for environmental samples was demonstrated by applying large volume injection (100 μL) and AFFL-SPE-cLC-MS. The platform should be well-suited for reliable determination of compounds with isotope-labeled internal standard in water samples. As with larger bore LC-MS systems, the cLC-MS system is not free from matrix effects, calling for fine-tuning of separation methodology and using isotope-labeled internal standards for each analyte.

## Author contributions

HR planned and supervised experiments, performed experiments and prepared/revised/ approved the manuscript. SA planned experiments, performed experiments and prepared/revised/approved the manuscript. DeD and DiD planned experiments, performed experiments and approved the manuscript. SFA performed experiments and approved the manuscript. SW and VB planned experiments and prepared/revised/approved the manuscript. EL planned and supervised experiments and prepared/revised/ approved the manuscript.

### Conflict of interest statement

The authors declare that the research was conducted in the absence of any commercial or financial relationships that could be construed as a potential conflict of interest.
