# Supplementary material for: Rugged Large Volume Injection for Sensitive Capillary LC-MS Environmental Monitoring
Source: Front Chem. 2017 Aug 28;5:62. doi: 10.3389/fchem.2017.00062 (PMC5581315; doi:10.3389/fchem.2017.00062)
Supplement: Supplementary file 1 [file DataSheet1.DOCX]

Supplementary material

**Rugged large volume injection for sensitive capillary LC-MS environmental monitoring**

Hanne Roberg-Larsen^1*^, Silvija Abele^1,2,^ ,Deniz Demir^1^, Diana Dzabijeva^2^, Sunniva Furre Amundsen^1^, Steven Ray Wilson^1^, Vadims Bartkevics^2,^ , Elsa Lundanes^1^

^1^ University of Oslo, Department of Chemistry, P.O. Box 1033, Blindern, NO-0315 Oslo, Norway

^2^ University of Latvia, Faculty of Chemistry, Jelgavas iela 1, LV-1004, Riga, Latvia

**Running title: Rugged large volume injection for environmental monitoring**

*Corresponding author:

Hanne Roberg-Larsen. Email: [Hanne.roberg-larsen@kjemi.uio.no](mailto:Hanne.roberg-larsen@kjemi.uio.no)

Tel.: +47 900 20 101

Table S 1 Analyte structure and SRM transitions used for quantification and identification.

|  |  |  | QExactive Orbitrap | | TSQ Quantiva triple Q. | | | | | | | | | | | | | |  |
| --- | --- | --- | --- | --- | --- | --- | --- | --- | --- | --- | --- | --- | --- | --- | --- | --- | --- | --- | --- |
| Analyte | Structure | Parent ion (*m/z*) | NCE | Product ion (*m/z*)  Q=qualifier | Product ion (m/z) | | | | Collision energy (V) | | | | RF- lense (V) | | | | |  |  |
| Sulfamethoxazole |  | 254.06 | 30 | 156.011 (Q) | 155.98 (Q) | | 23 | | | | | 67 | | | | | |  |  |
|  |  |  |  | 188.082 | 108.04 | | 33 | | | | | 67 | | | | | |  |  |
| Trimethoprim |  | 291.15 | 45 | 275.113 (Q) | 230.140 (Q) | | 31 | | | | | 85 | | | | | |  |  |
|  |  |  |  | 261.097 | 260.999 | | 34 | | | | | 85 | | | | | |  |  |
| Atenolol |  | 267.17 | 35 | 190.086( Q) | 145.130 (Q) | | 34 | | | | | 72 | | | | | |  |  |
|  |  |  |  | 225.123 | 190.064 | | 23 | | | | | 72 | | | | | |  |  |
| Azithromycin |  | 375.26 | 15 | 591.422(Q) | Not used | |  | | | | |  | | | | | |  |  |
| Clarithromycin |  | 748.49 | 15 | 590.391(Q) | Not used | | | | | | - | | | | | | - | | |
|  |  |  |  | 558.364 | Not used | | | | | | - | | | | | | - | | |
| Propanolol |  | 260.10 | 30 | 218.118(Q) | Not used | | | | | | - | | | | | | - | | |
|  |  |  |  | 183.081 | Not used | | | | | | - | | | | | | - | | |
| Diclofenac |  | 296.02 | 10 | 278.014(Q) | Not used | | | | - | | | | | | | - | | |  |
|  |  |  |  | 250.019 | Not used | | | | -- | | | | | | | - | | |  |
| Sulfapyridine |  | 250.06 | 35 | 156.011(Q) | 184.050 (Q) | | | | 24 | | | | | | | 67 | | |  |
|  |  |  |  | 184.086 | 155.955 | | | | 25 | | | | | | | 67 | | |  |
| Atenolol-d_7_ |  | 274.34 | Not used | - | | 145.099 (Q) | | 38 | | | | | | | 70 | | | |  |
|  |  |  |  |  |  | 190.124 | | 26 | | | | | | | 70 | | | |  |
| Sulfamethoxazole-(phenyl-^13^C_6_) | 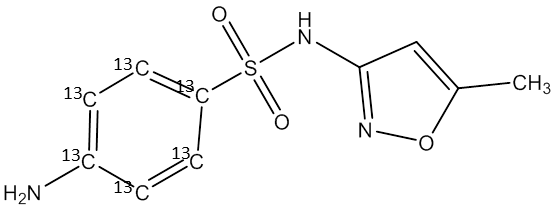 | 260.08 | Not used | - | 114.03 | | | | | 34 | | | | 64 | | | | |  |
|  |  |  |  |  | 161.96 | | | | | 23 | | | | 64 | | | | |  |

**Calculation of Asymmetry factor (As)**

Asymmetry was calculated at 10 % peak height. Asymmetry factors are shown in table S 2.


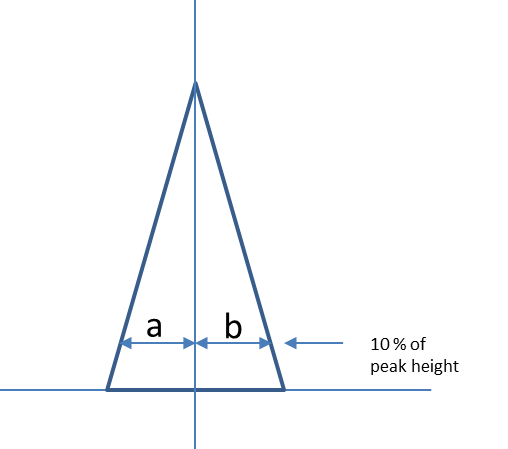

$$A_{s}=\frac{b}{a}$$

Table S 2 LOD and asymmetry factor for all analytes

| Analyte | LOD (ng/L) | Asymmetry factor |
| --- | --- | --- |
| Sulfamethoxazole | 0.05 | 1 |
| Trimethoprim | 0.05 | 1.2 |
| Atenolol | 0.5 | 1.5 |
| Azithromycin | 0.5 | 1.4 |
| Clarithromycin | 0.5 | 1 |
| Propanolol | 0.5 | 0.9 |
| Diclofenac | 10 | 0.7 |
| Sulfapyridine | 12.5 | 3.1 |

Table S 3 Apparent recovery, linearity and repeatability in spiked creek water

| **Analyte** | **Apparent recovery (%)** | **R^2^** | **Within-day repeatability (n=6)** | | | **Between day repeatability (n=3)** | | |
| --- | --- | --- | --- | --- | --- | --- | --- | --- |
|  |  |  | Mean A/Ais | SD | RSD (%) | Mean A/Ais | SD | RSD (%) |
| **Atenolol** | | | | | | | | |
|  | 150 | 0.9980 |  |  |  |  |  |  |
| 10 ng/L |  |  | 0.49 | 0.03 | 5 | 0.52 | 0.02 | 4 |
| 50 ng/L |  |  | 3.23 | 0.15 | 5 | 3.23 | 0.23 | 7 |
| 100 ng/L |  |  | 6.36 | 0.15 | 2 | 6.4 | 0.94 | 15 |
| **Sulfapyridine** | | | | | | | | |
|  | 81 | 0.9957 |  |  |  |  |  |  |
| 10 ng/L |  |  | 0.015 | 0.003 | 17 | 0.0250 | 0.0005 | 2 |
| 50 ng/L |  |  | 0.065 | 0.011 | 17 | 0.070 | 0.0026 | 4 |
| 100 ng/L |  |  | 0.113 | 0.013 | 11 | 0.133 | 0.0011 | 8 |
| **Sulfamethoxazole** | | | | | | | | |
|  | 151 | 0.9992 |  |  |  |  |  |  |
| 10 ng/L |  |  | 0.29 | 0.03 | 9 | 0.33 | 0.03 | 9 |
| 50 ng/L |  |  | 1.44 | 0.05 | 3 | 1.51 | 0.11 | 7 |
| 100 ng/L |  |  | 2.83 | 0.20 | 7 | 3.06 | 0.52 | 17 |
| **Trimethoprime** | | | | | | | | |
|  | 127 | 0.9928 |  |  |  |  |  |  |
| 10 ng/L |  |  | 0.583 | 0.06 | 10 | 0.7 | 0.3 | 46 |
| 50 ng/L |  |  | 2.12 | 0.14 | 6 | 2.5 | 3 | 105 |
| 100 ng/L |  |  | 4.22 | 0.29 | 7 | 4.7 | 4.4 | 93 |
|  |  |  |  |  |  |  |  |  |
